# MicroRNAs 125a and 125b inhibit ovarian cancer cells through post-transcriptional inactivation of EIF4EBP1

**DOI:** 10.18632/oncotarget.6474

**Published:** 2015-12-05

**Authors:** Maria Lee, Eun Jae Kim, Myung Jae Jeon

**Affiliations:** ^1^ Department of Obstetrics and Gynecology, Seoul National University College of Medicine, Seoul, Korea

**Keywords:** microRNA, microRNA 125a, microRNA 125b, epithelial ovarian cancer, EIF4EBP1

## Abstract

The aim of the present study was to identify the specific miRNAs involved in regulation of EIF4EBP1 expression in ovarian cancer and to define their biological function. miRNA mimics and miRNA inhibitors were used in quantitative PCR, western blotting, and luciferase reporter assays to assess cell migration, invasiveness, and viability. miR-125a and miR-125b were downregulated in ovarian cancer tissue and cell lines relative to healthy controls. Increased expression of miR-125a and miR-125b inhibited invasion and migration of SKOV3 and OVCAR-429 ovarian cancer cells and was associated with a decrease in EIF4EBP1 expression. The inverse relationship between miR-125a and miR-125b was corroborated by cotransfection of a luciferase reporter plasmid. Furthermore, miR-125a and miR-125b caused apoptosis and decreased cell viability and migration in an apparently EIF4EBP1-directed manner. Collectively, these results indicate that miR-125a and miR-125b are important posttranscriptional regulators of EIF4EBP1 expression, providing rationale for new therapeutic approaches to suppress tumour invasion and migration using miR-125a, miR-125b, or their mimics for the treatment of ovarian cancer.

## INTRODUCTION

Ovarian cancer is the fifth-leading cause of death from gynaecological malignancies [1]. Although the molecular genetics of its initiation and progression are not as well-described as other cancers, it is clear that translation initiation is deregulated during tumourigenesis [2]. This deregulation could be attributed to eukaryotic translation initiation factor 4E (EIF4E), an oncogene that regulates the translation of a specific subset of tumour-promoting mRNAs [2, 3]. Like other oncogenes, such as Akt and Ras, EIF4E induces senescence and acts as an intrinsic barrier to cancer [4, 5]. EIF4E-binding protein 1 (EIF4EBP1) plays a critical role in the control of protein synthesis and cell growth and survival, thus promoting tumourigenesis [6, 7]. Cells lacking EIF4EBP1s undergo p53-dependent senescence and are resistant to oncogenic transformation [8]. However, it is unclear how *EIF4EBP1* transcripts are regulated in ovarian cancer.

MicroRNAs (miRNAs) are small, endogenous RNA molecules that play important regulatory roles by targeting mRNAs for cleavage or translational repression [9, 10]. Although miRNA genes represent approximately 1% of the predicted genes in the genome, approximately 30% of protein-encoding genes are regulated by at least one miRNA [11, 12]. miRNAs play key roles in diverse pathways, including those involved in developmental processes and cell growth, differentiation, and apoptosis [11, 13, 14]. In ovarian cancers, some miRNAs are positively associated with malignancy, including facets such as tumour progression and chemotherapy resistance [15-19]. However, the full regulatory landscape of miRNAs in the pathogenesis of ovarian cancer has not been fully addressed.

Thus, we postulated that aberrantly-expressed miRNAs—whether over-expressed tumorigenic miRNAs or under-expressed protective miRNAs—contribute to the development of ovarian cancer by upregulating EIF4EBP1 expression. The aim of the present study was to identify the specific miRNAs involved in EIF4EBP1 expression in ovarian cancer cells and to define their functional effects.

## RESULTS

### Expression of miR-125a and miR-125b is significantly decreased in ovarian cancer tissue and cell lines compared to normal ovarian tissue

We compared miRNA expression profiles in ovarian cancer cell lines and human ovarian surface epithelial (HOSE) cell lines using microarray analysis (data not shown). In an effort to identify specific miRNAs that might regulate EIF4EBP1, we used the biocomputational prediction algorithms of three different programs (miRanda, TargetScan, and PicTar). This approach is known to provide a good balance of sensitivity and specificity [20]. Potential regulatory relationships with *EIF4EBP1* mRNA were identified for 15 miRNAs. Of these, the two most notable were miR-125a and miR-125b, which were significantly downregulated in ovarian cancer relative to HOSE cells on microarray analysis. Alignment of the 3′-UTR of *EIF4EBP1* revealed that the putative target sequences for miR-125a and miR-125b are highly conserved across mammalian species.

**Figure 1:**
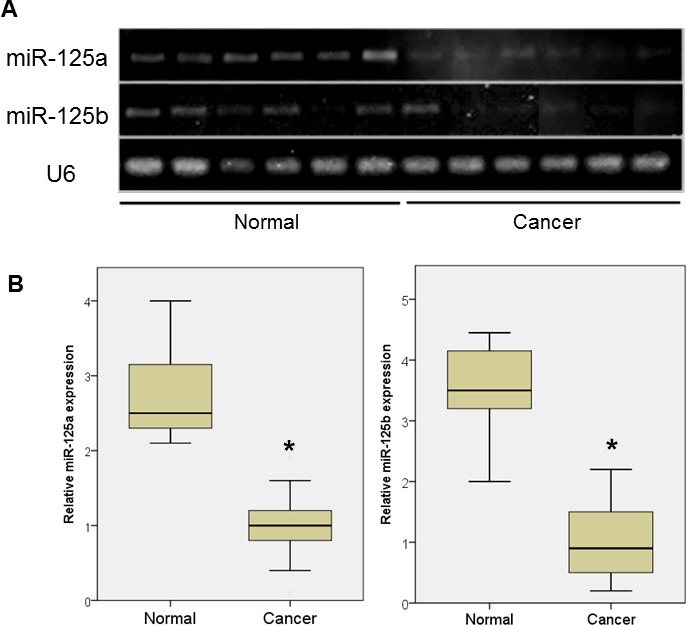

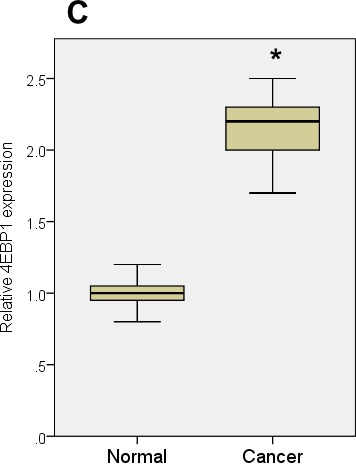

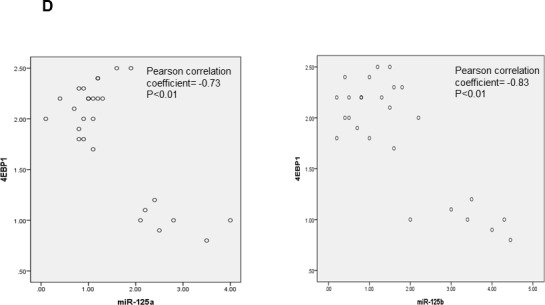
miRNA expression in ovarian cancer tissue and normal ovarian epithelial tissue **A.** qPCR analyses of two miRNAs in normal ovarian tissue and cancer tissue. **B.** Expression of miR-125a and miR-125b was significantly decreased in women with ovarian cancer (*n* = 20) compared to controls (*n* = 7). **C.** *4EBP1* mRNA expression was significantly increased in ovarian cancer patients compared with controls. **D.** An inverse correlation was observed between the expression levels of miR-125a or miR-125b and *EIF4EBP1* mRNA. Quantitative data representing the mean ± standard deviation (SD) are presented in the bar graph. *P < 0.01 compared with control expression levels.

The downregulation of miR-125a and miR-125b was also observed in ovarian cancer patients (Figure 1A, 1B), accompanied by a significant increase in *EIF4EBP1* mRNA expression (Figure 1C). The role of miR-125a and miR-125b as an inhibitor of EIF4EBP1 was further suggested by a significant, inverse correlation between the expression levels of miR-125a and miR-125b, and *EIF4EBP1* mRNA (Pearson correlation coefficient = −0.73 and −0.83, respectively; *p* < 0.01; Figure 1D).

**Figure 2:**
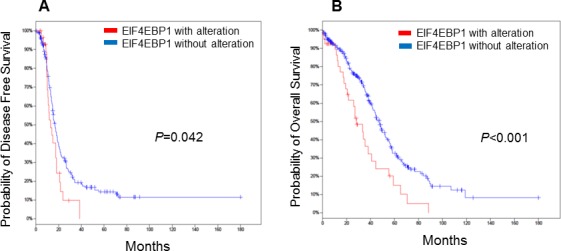
Kaplan-Meier plots for epithelial ovarian cancer patients stratified according to EIF4EBP1 expression Patients whose tumours showed *EIF4EBP1* alteration had significantly worse disease-free survival **A.** (*P* = 0.042) and overall survival **B.** (*P* = < 0.001).

We next examined the relationship between EIF4EBP1 expression and outcome. We searched high-grade serous epithelial ovarian carcinoma (HGS EOC) cases in The Cancer Genome Atlas (TCGA) for cases with *EIF4EBP1* alterations using cBioPortal [21]. Overall, 316 ovarian cancers with genome-wide gene expression data were available. In keeping with our *in vitro* and *in vivo* results, we found that *EIF4EBP1* mRNA expression was significantly higher in ovarian cancer tissue than in normal ovarian surface epithelium (*P* < 0.001). Furthermore, patients whose tumours exhibited *EIF4EBP1* expression alteration had significantly poorer disease-free survival (Figure 2A; *P* = 0.042) and overall survival (Figure 2B; *P* < 0.001).

### Both miR-125a and miR-125b inhibit EIF4EBP1 mRNA and protein levels

**Figure 3:**
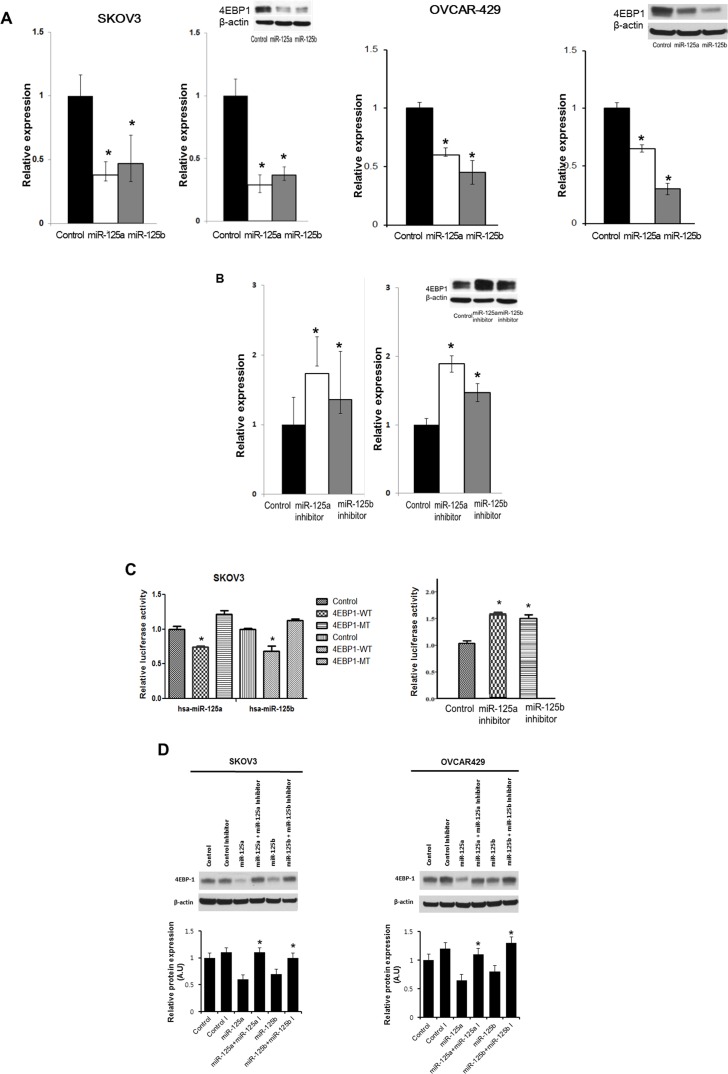
Effects of overexpression and inhibition of miR-125a and miR-125b and co-transfection of miR-125a or miR-125b and miR-125a or miR-125b inhibitors on 4EBP1 expression qRT-PCR analysis of miR-125 and miR-125b levels in SKOV3 and OVCAR-429 cells transfected with specific miRNA mimic (**A.**, left) or miRNA inhibitor (**B.**, left). Transfection with miR-125a or miR-125b mimic decreased 4EBP1 mRNA and protein levels (**A.**, right). Conversely, transfection with miR-125a or -125b inhibitor resulted in an increase in 4EBP1 levels (**B.**, right). miR-125a or -125b overexpression significantly decreased the relative luciferase activity of the wild-type 3′-UTR, but not of the mutant 3′-UTR in which the miRNA-binding sites were deleted (**C.**, left). Conversely, treatment of miR-125a or -125b inhibitors significantly increased the relative luciferase activity (**C.**, right). Co-transfection of miR-125a or miR-125b and miR-125a or miR-125b inhibitors in SKOV3 and OVCAR-429 cells enhanced 4EBP1 protein levels **D.**. Each experiment was conducted in triplicate. Quantitative data representing the mean ± SD are presented in the bar graph. *P < 0.01 compared with negative controls.

We performed a series of functional studies to determine the roles of miR-125a and miR-125b in the regulation of EIF4EBP1. First, using specific miR mimics, we investigated whether overexpression of miR-125a or miR-125b was sufficient to reduce EIF4EBP1 levels in SKOV3 and OVCAR-429 ovarian cancer cells. The miR-125a and miR-125b mimics repressed *EIF4EBP1* mRNA and protein levels in both cancer cell lines (Figure 3A). Next, cultured SKOV3 cells were transfected with a miR-125a inhibitor, a miR-125b inhibitor, or a negative control. Treatment with an inhibitor of miR-125a or miR-125b enhanced *EIF4EBP1* mRNA and protein levels (Figure 3B).

### EIF4EBP1 is a direct target of miR-125a and miR-125b

To assess whether miR-125a and miR-125b directly alter EIF4EBP1 expression, we transfected SKOV3 cells with luciferase expression plasmids containing the full-length wild-type 3′-UTR of the *EIF4EBP1* transcript or the 3′-UTR in which the miR-125a and miR-125b binding sites had been deleted. Co-transfection of the plasmid containing the wild-type UTR plus a miR-125a or miR-125b mimic, but not a negative control, resulted in a significant decrease in relative luciferase activity (Figure 3C). In contrast, co-transfection of the plasmid with the mutated UTR plus either mimic completely abolished miR-125a and miR-125b-mediated repression, demonstrating the specificity of these miRNAs for EIF4EBP1 suppression.

Because miR-125a and miR-125b mimics reduced *EIF4EBP1* 3′-UTR-driven luciferase activity, inhibitors of these two miRNAs were assessed to determine if they exerted the opposite effect. Co-transfection with the miRNA mimics plus a miR-125a or miR-125b inhibitor significantly increased the luciferase activity relative to controls (Figure 3C). Taken together, these results show that both miR-125a and miR-125b can directly influence *EIF4EBP1* through specific binding to its 3′-UTR. Furthermore, co-treatment with miR-125a or miR-125b plus their inhibitors enhanced *EIF4EBP1* mRNA and protein levels (Figure 3D). Taken together, these results show that both mi miR-125a and miR-125b can directly influence *EIF4EBP1* through specific binding to its 3′-UTR.

### miR-125a and miR-125b mimics decrease cell invasion and migration in EOC cells

**Figure 4:**
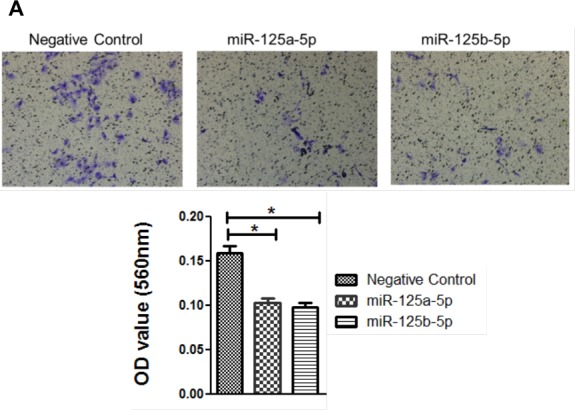

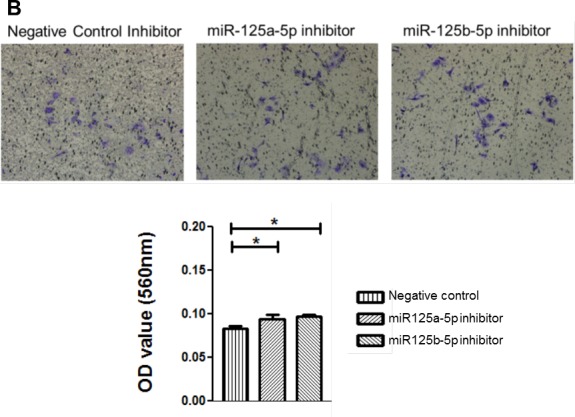

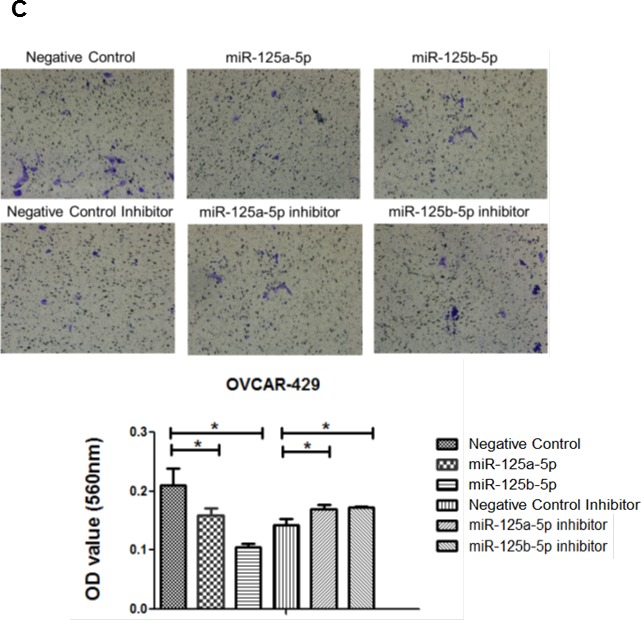

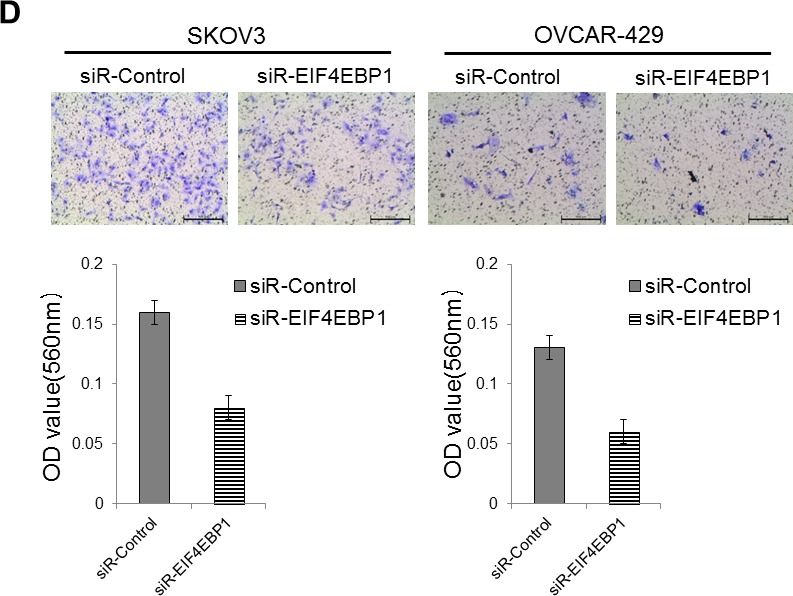

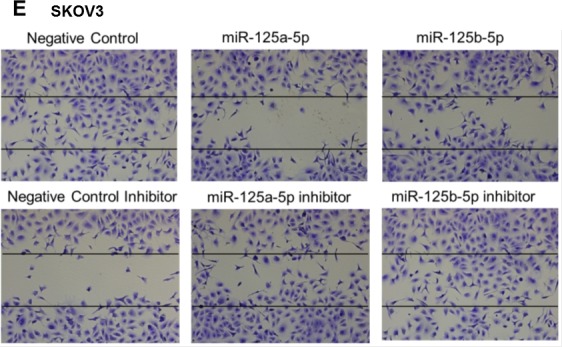

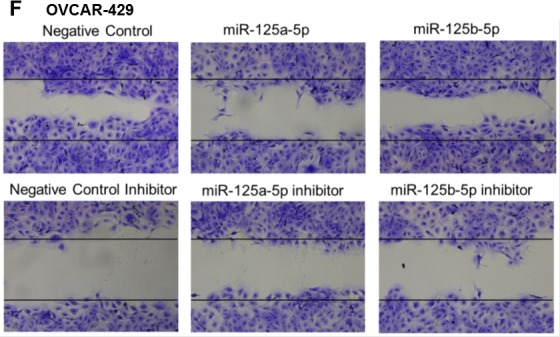

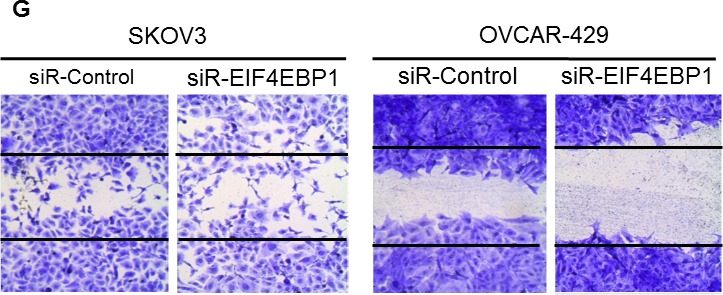

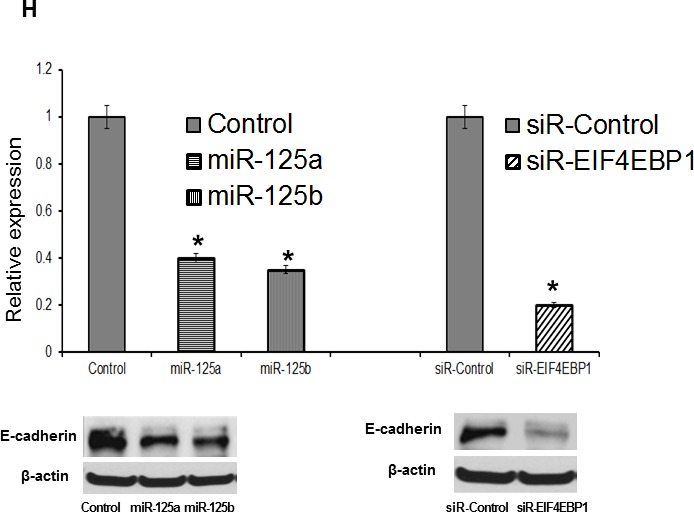
Invasion and wound healing assays in miR-125a, miR-125b-treated, and knockdown of EIF4EBP1 in SKOV3 and OVCAR-429 ovarian cancer cells Treatment with miR-125a and miR-125b mimics resulted in decreased invasion compared to controls **A., C.**. In contrast, treatment with miR-125a and miR-125b inhibitors increased invasion by SKOV3 and OVCAR-429 cells **B., C.**. In EIF4EBP1 siRNA-treated cells, cell invasion was decreased compared to controls **D.**. Treatment with miR-125a and miR-125b mimics resulted in decreased motility of SKOV3 and OVCAR-429 cells compared to control cells **E., F.**. In contrast, treatment with miR-125a and miR-125b inhibitor increased cell motility **E., F.**. In EIF4EBP1 siRNA-treated cells, cell motility was decreased compared to controls **G.**. E-cadherin expression was decreased in miR-125a- and miR-125b-treated EOC cells and siR-EIF4EBP1 cells compared to controls **H.**. *P < 0.01 compared with negative controls.

An invasion assay was performed to evaluate whether EIF4EBP1 regulation by miR-125a and miR-125b prevents invasion in EOC cells. To confirm the possible role of miR-125a and miR-125b in cell invasion, we evaluated the invasive potential of SKOV3 and OVCAR-429 ovarian cancer cells treated with mimics of these miRNAs. Indeed, treatment with miR-125a and miR-125b mimics decreased invasion relative to control treatment (Figure 4A, 4C). In contrast, miR-125a and miR-125b inhibitor treatment increased invasion by these cells (Figure 4B, 4C).

We next assessed the impact of EIF4EBP1-silencing by RNA interference on invasion in SKOV3 and OVCAR-429 cells. Diminished invasion abilities were present in siRNA-treated SKOV3 and OVCAR-429 cells (Figure 4D).

To assess whether EIF4EBP1 plays a role in migration inhibited by miR-125a and miR-125b in EOC cells, we conducted a wound-healing assay to assess cell motility. Treatment with miR-125a and miR-125b mimics resulted in decreased motility of SKOV3 and OVCAR-429 cells compared to control treatment. In contrast, miR-125a and miR-125b inhibitor treatment increased cell motility of these cells (Figure 4E, 4F). Similarly, EIF4EBP1-knockdown inhibited cell motility in SKOV3 and OVCAR-429 cells (Figure 4G). E-cadherin expression was decreased in miR-125a- and miR-125b-mimic-treated EOC cells and in siR-EIF4EBP1-treated SKOV3 and OVCAR-429 cells relative to control cells (Figure 4H). Taken together, these results demonstrate that miR-125a- and miR-125b-mediated suppression of EIF4EBP1 plays an important role in cell invasion and mobility in ovarian cancer.

### miR-125a or miR-125b inhibits ovarian cancer cell proliferation by repressing EIF4EBP1 expression

**Figure 5:**
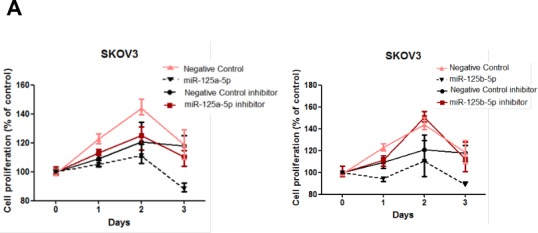

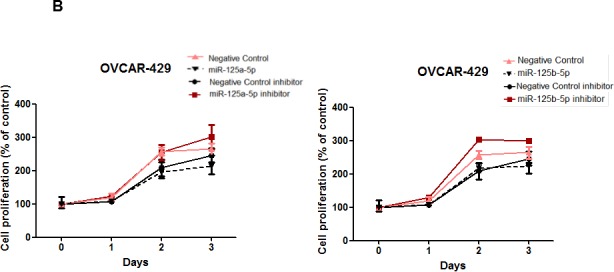

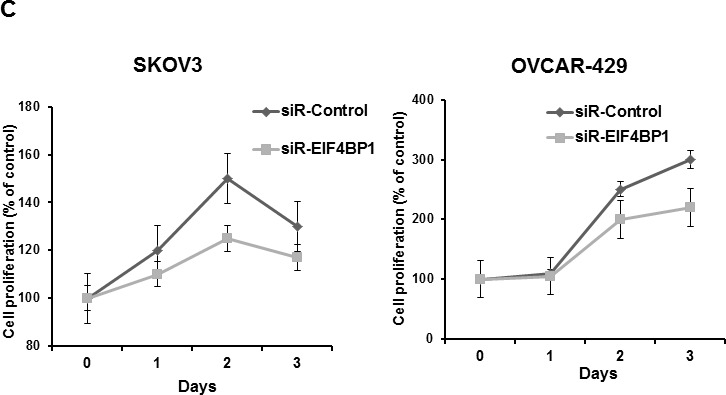

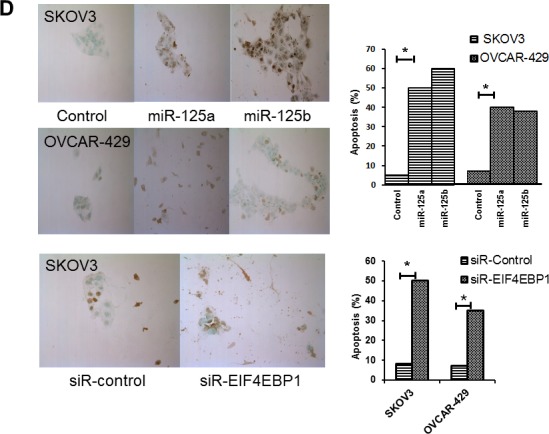
Cell proliferation after treatment with miR-125a or miR-125b, their inhibitors, or knockdown of EIF4EBP1 miR-125a or miR-125b overexpression significantly decreased ovarian cancer cell proliferation 48 to 72 h after transfection. Treatment with miR-125a or miR-125b inhibitor increased viability and proliferation of SKOV3 and OVCAR-429 cells **A., B.**. In EIF4EBP1 siRNA-treated cells, cell proliferation was decreased compared to controls **C.** TUNEL assay revealed that overexpression of miR-125a or miR-125b and knockdown of EIF4EBP1 increased apoptosis in these cells, as indicated by the intense dark brown staining of the majority of SKOV3 and OVCAR-429 cells compared to control cells. Annexin V/PI-based flow cytometric analysis was conducted to check apoptotic cell death by miR-125a, miR-125b, or knockdown of EIF4EBP1 in ovarian cancer cells 24 h after treatment **D.**. *P < 0.01 compared with negative controls.

In addition to their role in reducing invasive and migratory abilities of ovarian cancer cells, we assessed the role of miR-125a and miR-125b in cellular proliferation in SKOV3 and OVCAR-429 cells. miR-125a and miR-125b overexpression significantly decreased proliferation of ovarian cancer cells within 48 to 72 h of treatment (Figure 5A, 5B), as did the knockdown of EIF4EBP1 (Figure 5C). Conversely, treatment with a miR-125a or miR-125b inhibitor increased proliferation (Figure 5A, 5B).

To explore the possibility that suppression of cell growth by miRNA-mediated downregulation of EIF4EBP1 expression was caused by apoptotic cell death, we measured the levels of apoptosis in SKOV3 and OVCAR-429 cells stably transfected with miR-125a or miR-125b, and in control cells. TUNEL assay revealed that overexpression of miR-125a or miR-125b increased the rate of apoptosis relative to control cells (Figure 5D). Cellular apoptosis was also increased in EIF4EBP-knockdown cells relative to the siRNA-control group (Figure 5D).

### miR-125a and miR-125b regulates the expression of EIF4EBP1 and VEGF

**Figure 6:**
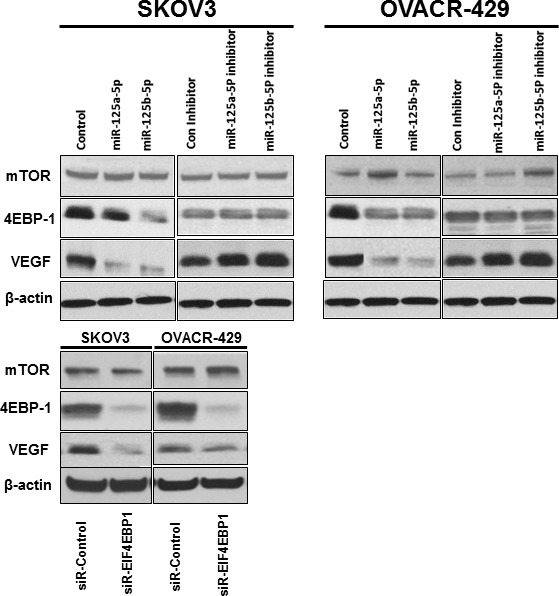
Effects of overexpression and inhibition of miR-125a or miR-125b and EIF4EBP1 knockdown on expression of 4EBP1, VEGF, and mTOR VEGF expression was downregulated in miR-125a- or miR-125b-overexpressing and EIF4EBP1 knockdown SKOV3 and OVCAR-429 cells. However, no significant difference in mTOR expression was observed between miR-125a- or miR-125b-overexpressing cells and cells in which miR-125a or miR-125b was inhibited. In knockdown of EIF4EBP1 cells, no significant difference in mTOR expression was observed relative to controls.

To explore the molecular mechanism underlying miR-125a or miR-125b inhibition of cell viability and proliferation, we examined the expression levels of mTOR and VEGF, as well as EIF4EBP1, by western blotting (Figure 6). Interestingly, VEGF expression was markedly downregulated in miR-125a or miR-125b-overexpressing SKOV3 and OVCAR-429 cells. However, no significant difference in mTOR expression was observed between either the cells overexpressing miR-125a or miR-125b, or the cells treated with miR-125a or miR-125b inhibitor and their respective control cells. These results were consistent with those of EIF4EBP1-knockdown cells (Figure 6).

## DISCUSSION

In this study, we elucidated the post-transcriptional inhibition of EIF4EBP1-mediated pathways in ovarian cancer cells by the miRNAs miR-125a and miR-125b. We showed a direct interaction between miR-125a, miR-125b, and EIF4EBP1; overexpression of miR-125a and miR-125b was associated with suppression of luciferase activity under control of the *EIF4EBP1* 3′-UTR. Furthermore, we demonstrated that overexpression of miR-125a and miR-125b caused significant downregulation of both *EIF4EBP1* mRNA and protein.

Translation factors functionally interact with oncogenes and underlie most human cancers. The increased or reduced expression of these factors is associated with the development of specific cancers and crucially affects disease progression. eIF4E mediates phenotypic changes by selectively enhancing translation of a limited pool of mRNAs that code for proteins involved in malignancy. As such, enhanced eIF4E can contribute to every aspect of malignant progression [4]. EIF4EBP1, a protein that can inhibit the translation initiation of capped mRNAs, is a downstream target of the AKT/mTOR pathway, and when EIF4EBP1 is phosphorylated, its inhibitory impacts decrease, allowing increased translation initiation [6]. Indeed, in a previous study at our institution, an elevated level of p-eIF4EBP1 was associated with advanced stage, higher histologic grade, shorter disease-free survival rate, and chemoresistance [22]. The increase in *eIF4EBP1* mRNA is somewhat surprising because such an increase would inhibit translation of capped mRNAs that can promote tumour progression.

miR-125a is a tumour-suppressing miRNA downregulated in several cancers [23-27]. In the present study, we found that miR-125a was downregulated in ovarian cancer tissue relative to normal tissue. Similarly, another member of the miR-125a family, the isoform miR-125a-3p, is downregulated in non-small cell lung cancer cells; its expression is negatively correlated with pathological stage and metastasis [30]. miR-125a-3p is also suspected to regulate genes encoding chemokine ligand 4 (*CCL4*) and *IGF2*. Both promote tumour cell migration, implying a role for miR-125a-3p in tumour suppression [30-32]. In the current study, miR-125b was also downregulated in ovarian cancer tissue. Overexpression of miR-125b inhibits tumour-induced angiogenesis associated with HER2 and HER3 expression in ovarian cancer cells [33]. On the other hand, poly-A binding protein-1 (PAPB-1), known to promote translation of capped mRNAs, may also be targeted by miR-125b [34]. A previous study reported that loss of miR-125b is associated with increased EIF4EBP1 protein levels in breast cancers [35].

EIF4EBP1 was significantly elevated in ovarian cancer cells relative to control cells. *EIF4EBP1* mRNA levels were also elevated. Our results indicated that EIF4EBP1 was a direct target of miR-125a and miR-125b, and that reduction of miR-125a and miR-125b levels using miR-125a and miR-125b inhibitors partially restored EIF4EBP1 expression, as well as invasiveness and migratory capabilities in SKOV3 and OVCAR429 cells. EIF4EBP1 siRNA decreased cell proliferation and invasion in SKOV3 and OVCAR429 cells, indicating that these abilities were EIF4EBP1-dependent.

In case studies, *EIF4EBP1* alteration was strongly related to survival in ovarian cancer patients. In keeping with our *in vitro* findings, we found that EIF4EBP1 expression was significantly elevated in ovarian cancer tissue and cell lines and that protein levels were strongly associated with prognosis of patients with ovarian cancer. Moreover, miR-125a and miR-125b downregulates VEGF and EIF4EBP1 levels, suggesting that suppression of these tumorigenic targets may be the mechanism by which these miRNAs suppress tumour growth.

In conclusion, we found that miR-125a and miR-125b were frequently downregulated in ovarian cancer cells. Ectopic expression of miR-125a and miR-125b inhibited ovarian cancer cell proliferation, migration, and invasion *in vitro.* Further experiments revealed that EIF4EBP1 was a direct and functional target of miR-125a and miR-125b in ovarian cancer cells. Our functional analysis of these two miRNAs, therefore, suggests that miR-125a and miR-125b may represent both diagnostic markers and therapeutic targets in the treatment of ovarian cancer patients.

## MATERIALS AND METHODS

### Tissue collection

All experiments were performed with the approval of the review board for human research of Seoul National University Hospital. Samples were collected between July 2012 and July 2014 from 20 ovarian cancer patients who underwent surgery and a control group of seven patients with benign gynaecologic disease. Informed consent was obtained from all of the participating women. Ovarian tissue samples 1 × 1 cm in size were collected at the time of surgery. The samples were immediately snap-frozen in liquid nitrogen and kept at −80°C until RNA extraction was performed.

### Cell culture

SKOV3 and OVCAR429 ovarian cancer cell lines were purchased from Korean Cell Line Bank (KCLB, Seoul, Korea) and six types of ovarian cancer cell lines (SNU840, OVCAR3, TOV112D, YDOV-151, YDOV-161 and YDOV-139) were provided by Korea Gynecologic Cancer Bank through Bio & Medical Technology Development Program of the MSIP, Korea. Cell lines were maintained in Dulbecco's modified Eagle medium (DMEM; Sigma-Aldrich, St Louis, MO, USA) at 37°C in an atmosphere of 5% CO_2_. Culture medium was replaced with fresh medium every 2-3 days. Cells were used between passages 5 and 10.

### Transfection of miRNAs

SKOV3 and OVCAR429 cells were plated at a density of 5 × 10^5^ cells per 100-mm culture dish, cultured in DMEM containing 10% foetal bovine serum (FBS) without antibiotic or antimycotic, and incubated at 37°C in a humidified atmosphere of 5% CO_2_ until the cells reached 40-50% confluence. Cells were then transfected with synthetic precursor miRNA, miR-125a, or miR-125b at a final concentration of 100 nM using Lipofectamine 3000 (Invitrogen, Carlsbad, CA, USA). Forty-eight hours after transfection, cell lysates were collected for RNA or protein isolation. Expression levels based on reporter Cy3 fluorescence revealed high transfection efficiencies, exceeding 50% in some experiments.

### Knockdown of EIF4EBP1

Small interfering RNAs (siRNA) were used to inhibit endogenous *EIF4EBP1* in ovarian cancer cell lines. Scrambled siRNA (universal negative control siRNA), EIF4EBP1-siRNA was purchased (Applied Biosystems, Carlsbad, CA, USA), and the two siRNAs to each targeted gene were (EIF4EBP1 #1 sense: 5′CGAACCCUUCCUUCCGAAUtt-3′, antisense 5′AUUCGGAAGGAAGGGUUCGtt-3′; EIF4EBP1 #2 sense: 5′GAUCAUCUAUGACCGGAAAtt-3′, antisense 5′UUUCCGGUCAUAGAUGAUCct-3′). Cells were plated to 40-60% confluency and transfected with 10 nM EIF4EBP1-siRNA using Lipofectamin 3000 reagent (Invitrogen, Carlsbad, CA, USA) according to the manufacturer's instructions. Knockdown efficiency was confirmed by RT-qPCR. The transfected cells were used for various assays 48-72 h after transfection to allow for the effective knockdown of EIF4EBP1.

### Quantitative real-time PCR analysis (qPCR)

Total RNA was extracted from cell lines and tissue samples using the mirVana miRNA Isolation Kit (Ambion, Austin, TX, USA). cDNA was generated using the GoScript Reverse Transcription system (Promega, Madison, WI, USA) according to the manufacturer's protocol. PCR amplification was carried out with the following primers: *EIF4EBP1*, forward 5′-ATGTCCGGG GGCAGCAGCTGCAGCCAG-3′ and reverse 5′-ACAGGUGAGGUUCUUGGGAACU-3′; and *GAPDH*, forward 5′-GTCGGAGTCAACGGATTTGG-3′ and reverse 5′-AAAAGCAGCCC TGGTGACC-3′. Reaction mixtures were prepared using the SYBR Premix Ex Taq (TaKaRa, Tokyo, Japan) according to the manufacturer's protocol. Each 20-μL reaction mixture contained10 μL 2× SYBR Premix Ex Taq, 0.4 μL 50× ROX Reference Dye, 4 μL 10 μM forward and reverse primer mixture, 3.6 μL nuclease-free water, and 2 μL cDNA. PCR was carried out under conditions of 95°C for 5 sec followed by 40 cycles of denaturation at 95°C for 5 sec, annealing at 60°C for 34 sec, and extension at 95°C for 15 sec, 60°C for 1 min, and 95°C for 15 sec. Reverse transcription and miRNA quantification were carried out using TaqMan miRNA Assays (Applied Biosystems, Carlsbad, CA, USA). PCR amplification was conducted using the TaqMan Universal PCR Master Mix according to the manufacturer's protocol. The samples were analysed using the 7500 real-time PCR system (Applied Biosystems). All PCRs were performed in triplicate, and the specificity of each reaction was determined by melting curve analysis at the dissociation stage. For relative quantification, the qPCR data were analysed using the 2^−ΔΔCt^ method, where β-actin and U6B were used as internal controls for *EIF4EBP1* and miRNAs, respectively.

### Western blot analysis

Cells were lysed in 200 μl RIPA buffer (150 mM sodium chloride, 1% NP 40, 0.5% sodium deoxycholate, 0.1% sodium dodecyl sulphate, 50 mM Tris-HCl [pH 8.0], 100 mM PMSF) and centrifuged at 14,000 *g* for 10 min at 4°C. The supernatant was mixed with denaturing sample buffer (1:1) and boiled for 5 min at 94°C. Equal amounts of protein (50 μg) were loaded and separated by 10% SDS-polyacrylamide gel electrophoresis and blotted onto nitrocellulose membranes (BioRad, Hercules, CA, USA). The membranes were blocked in Tris-buffered saline with Tween 20 (TBST) containing 5% non-fat dry milk for 1 h at 4°C and incubated with anti-4E-BP1 (1:2000), anti-phospho-4E-BP1 (1:1000), anti-mTOR (1:2000), anti-E-cadherin (1:1000) (all from Cell Signalling Technologies, Danvers, MA, USA), or anti-VEGF (1:2000; Santa Cruz Biotechnology, Santa Cruz, CA, USA) antibodies overnight at 4°C. Anti-β-actin antibody (1:5000; Sigma-Aldrich) was used as a control. Membranes were washed in TBST and incubated with horseradish peroxidase-conjugated secondary antibodies (Jackson Immunoresearch, West Grove, PA, USA) for 1 h at room temperature and washed again in TBST. The signal was detected using an enhanced chemiluminescence kit (Thermo Scientific, Rockford, IL, USA), and intensity was quantified using ImageJ software.

### Luciferase reporter assay

To validate the 4EBP1 3′-untranslated region (3′-UTR) as a target of miR-125a and miR-125b, *in vitro* assays used the miTarget miRNA 3′-UTR target clones (HmiT004676-MT01; Genecopoeia, Rockville, MD, USA). These miRNA target clones consisted of the pEZX-MT01 vector containing the coding sequences of both firefly and *Renilla* luciferase; the full 3′-UTR of the *EIF4EBP1* transcript (GenBank accession number: NM_004095) was inserted downstream of the firefly luciferase sequence. TargetScan (www.targetscan.org) predicted that the miR-125a and miR-125b binding sites are located at nucleotides 142 to 148. For mutagenesis assays, these two miRNA-binding sites within the 3′-UTR of the 4EBP1 transcript were deleted. After heat-shock transformation in competent *Escherichia coli* cells (One Shot TOP 10 competent cells; Invitrogen), the plasmids were amplified in Luria-Bertani medium supplemented with 50 μg/ml kanamycin (Bio Basic, Markham, ON, Canada). Plasmid DNA was prepared on columns (NucleoBond PC 500; Macherey-Nagel, Düren, Germany), and the identities of the amplified plasmids were confirmed by capillary sequencing (ABI 3730XL, Applied Biosystems) using the sequencing primers 5′-CUCACUCAGGGCACCUGC-3′ (forward) and 5′-UUCAAUCCCAGAGUCCCU-3′ (reverse). SKOV3 and OVCAR429 cells were plated at a density of 1 × 10^4^ cells per well in 96-well plates. A total of 100 ng plasmid DNA was co-transfected with miRNA mimic, miRNA inhibitors, or negative controls, as described above. Luciferase assays were performed 48 h after transfection using the Dual-Luciferase Reporter Assay System (Promega). Firefly luciferase activity was normalized to *Renilla* luciferase expression level for each sample. Each experiment was conducted in triplicate.

### Invasion and wound healing assays

Invasion by tumour cells was analysed using Cell Invasion Assay Kit (8-μm pore size; Chemicon, Billerica, MA, USA) according to the manufacturer's protocol. After transfection, cells were suspended in serum-free medium and plated at a density of 5 × 10^5^ cells per well in the upper chamber. The lower chamber was filled with culture medium supplemented with 10% FBS as the chemoattractant. After 48 h, the non-invading cells on the upper side of the membrane were gently removed with a cotton swab. The cells that had invaded the lower surface of the membrane were stained, air-dried, photographed, and counted under a light microscope. For quantification, the stained cells were dissolved with 10% acetic acid, and absorbance was measured at 560 nm. The assay was performed in triplicate.

The wound healing assay was performed with a Cytoselect 24-Well Cell Invasion Assay Kit (Cell Biolabs, San Diego, CA, USA) according to the manufacturer's protocol. Transfected cells were added to either side of the open end at the top of the insert. When the cells had formed a monolayer, the insert was removed to generate a consistent 0.9-mm gap wound in the middle. To determine migration distance, the size of the wound was measured at each time point. At multiple time points, cells were fixed and stained with methylene blue and photographed.

### Cell viability assay

An equal number of cells (1 × 10^4^) transfected with miR-125a and miR-125b were seeded in 96-well plates and incubated for 48 h. The number of viable cells was determined using a Cell Counting Kit (CCK; Dojindo, Kumamoto, Japan). CCK reagents were added to cultures and incubated for 2 h; measurement of absorbance of each well at 540 nm with a micro-ELISA reader (Molecular Devices; Sunnyvale, CA, USA) was then performed.

### TUNEL assay

An equal number of cells (5 × 10^3^) transfected with miR-125a and miR-125b were seeded in a Lab-Tek II Chamber Slide w/Cover RS Glass Slide Sterile and incubated for 48 h. The TUNEL assay was performed using the ApopTag Kit S7100 (Millipore, Billerica, MA, USA) according to the manufacturer's instructions. Colour development was carried out using a 3,3′-diaminobenzidine solution, and sections were counterstained with methyl green. Negative control sections, processed in the absence of terminal deoxynucleotidyl transferase, showed no staining. For measurement of apoptotic cell death in ovarian cancer cells, we performed flow cytometric analysis using Annexin-V and PI staining (BD Pharmingen, CA) according to the manufacturer's protocol.

### Statistical analysis

Statistical analyses were performed using SPSS 19.0 for Windows (SPSS, Chicago, IL, USA). The normality of the data was assessed using the Shapiro-Wilk test. Comparisons between two groups were performed using the two-sample *t*-test or the Mann-Whitney *U* test for continuous variables and the chi-square test for categorical variables. For comparisons among more than two groups, one-way analysis of variance was performed, and Dunnett's procedure was used for multiple comparisons. Correlation analyses were performed using Pearson's correlation analyses. All of the statistical tests were two-tailed, and p-values less than 0.05 were considered statistically significant.
